# Combined Effects of tDCS and Language/Cognitive Intervention on the Naming of Dementia Patients: A Systematic Review and Meta-Analysis

**Published:** 2020-05

**Authors:** Haewon BYEON

**Affiliations:** Department of Speech Language Pathology, College of Health Science, Honam University, Gwangju, Republic of Korea

**Keywords:** Transcranial direct current stimulation, Dementia, Naming ability, Meta-analysis

## Abstract

**Background:**

The evidence of transcranial direct current stimulation (tDCS) treatment effects on dementia is still insufficient. This study aimed to prove the scientific basis of tDCS by conducting a meta-analysis of previous studies that examined the effects of tDCS on the naming of patients with dementia.

**Methods:**

The literature search was conducted for publications published from Jan 2000 to Jun 2019 using four academic databases (PubMed, Web of Science, MEDLINE, and Cochrane Library). This study found 129 publications by searching academic databases according to the PRISMA protocol. Among them, irrelevant studies were excluded, and finally, 4 studies were meta-analyzed.

**Results:**

The score of the quality assessment on five studies ranged from 21 to 26 points, rated good or better. Meta-analysis results, tDCS had no significant effect on the improvement of naming ability of dementia. On the other hand, the effect size of the tDCS intervention + language/cognitive training was significant ‘large effect (SMD=0.72, 95% CI: 0.05, 1.39)’.

**Conclusion:**

This combination of tDCS and language/cognitive training significantly improved the naming ability of dementia patients.

## Introduction

More interest has been given to the quality of life in old age along with aging, interest in the prevention and treatment of dementia, a representative disease of old age, is also increasing.

It is impossible to completely cure Alzheimer’s dementia, but it has become possible to improve symptoms or delay the progress when it is detected and intervened early because a cognitive function enhancer that can maintain acetylcholine normally is developed (1, 2). Therefore, early detection and early intervention are very important tasks from the clinical viewpoint of dementia.

Naming is the distinguishing feature of dementia patients in the early stage and is a sensitive test index primarily used for screening dementia (3, 4). Naming requires the simultaneous action of cognitive abilities (e.g., attention, perception, and memory) and semantic language processing (e.g., lexical knowledge and phonology) (5). The flaws in naming are prominent from the onset of dementia (5).

Various treatments such as traditional cognitive training and computer-based training have been used to improve the naming ability of patients with neurological speech disorders and a previous meta-analysis study (6) proved the treatment effects of them. Recently, transcranial direct current stimulation (tDCS) has been applied as a method for improving the cognitive/language ability of dementia patients (7). tDCS is a non-invasive brain stimulation that activates the cerebral cortex by flowing minute DC current to the scalp and is a medical device developed based on the principle of neuroplasticity (7). It has been known that it enhances the linguistic ability of brain-damaged patients (8) without serious side effects (9).

Since 2010, numerous previous studies (10–15) have reported the effects of tDCS on the naming ability of patients with brain damage and these efforts have accumulated the scientific basis of tDCS. However, previous studies still have not provided enough scientific evidence for treatment because first, they were conducted primarily for patients with aphasia than dementia and second, they were mainly to experimental studies with a small sample size (16). In addition, the effectiveness of tDCS differs among studies. Costa et al. (10) reported that tDCS significantly improved language ability, while Westwood & Romani (17) showed that tDCS intervention did not affect the improvement of language ability significantly.

The evidence of tDCS’s treatment effects on dementia is still insufficient. This study aimed to prove the scientific basis of tDCS by conducting a meta-analysis of previous studies that examined the effects of tDCS on the naming of patients with dementia.

## Methods

This meta-analysis study was conducted through research question selection, systematic literature search and selection, evaluation of literature quality, data extraction and coding, data analysis, and result interpretation.

### Literature Search

The literature search was conducted for publications published from Jan 2000 to Jun 2019 using four academic databases (PubMed, Web of Science, MEDLINE, and Cochrane Library). The search terms ‘dementia’, ‘Alzheimer disease’, ‘Lewy bodies disease’, ‘frontotemporal dementia’, ‘primary progressive aphasia’, ‘mild cognitive impairment’ ‘transcranial direct current stimulation’, ‘tDCS’, ‘naming’, ‘generative naming’, ‘naming ability’, ‘confrontational naming’, ‘responsive naming’, ‘semantic fluency’, ‘verbal fluency’, ‘phonemic fluency’, ‘executive function’, ‘cognitive rehabilitation’, ‘cognitive training’, ‘language recovery’, and ‘language therapy’ were used.

The studies to be included in the analysis were selected by preparing Patient–Intervention–Comparison–Outcome–Study (PICOS) design according to the PRISMA protocol (18). The inclusion criteria of this study were first, studies conducted on dementia, second, experimental studies for examining the effects of tDCS, and third, academic publications published in English. Intervention studies using non-invasive brain stimulation or drugs, survey studies, and qualitative studies were excluded from this meta-analysis.

### Selection of Final Analysis Data

**Fig. 1:**
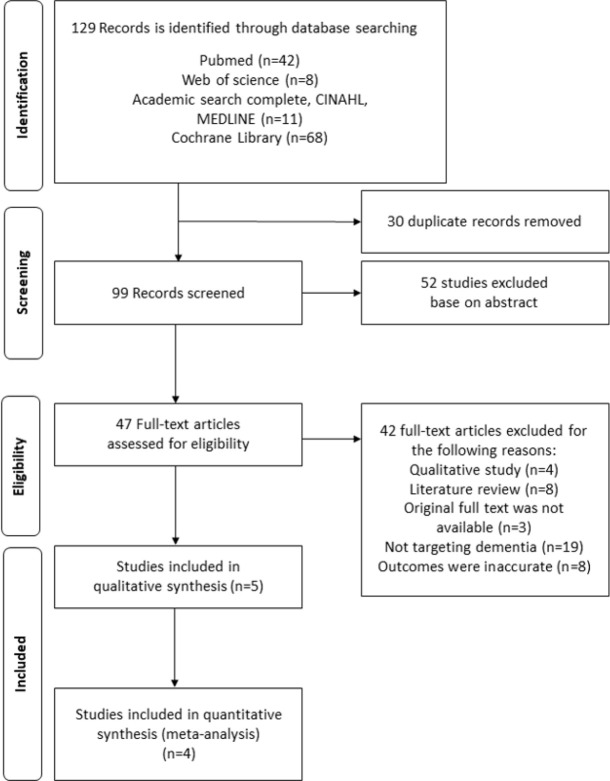
The flow diagram of study

This study found 129 publications by searching academic databases according to the PRISMA protocol. As a first screening step, the duplicated literature (n=30) was excluded and 52 studies, not related to the objective of this study based on the titles and abstracts of these studies, were also excluded. As a second step, the full texts of the remaining 47 papers were examined and excluded qualitative study (n=4), literature review (n=8), not targeting dementia (n=19), inaccurate outcomes (n=8), and no available original full text (n=3). Afterward, quality assessment was conducted for five studies. Among these five studies, one study that only presented the difference between pre- and post-values was excluded. As a result, the final four studies were used for the meta-analysis. The flow diagram of this search process is summarized in Fig. 1.

### Quality Assessments for Systematic Review

This study conducted a quality assessment using the “Standard Quality Assessment Criteria for Evaluating Primary Research Papers from a Variety of Fields” (19). It is an evaluation tool that can confirm the methodological quality by evaluating 14 items (i.e., study objective, study design, subject selection, subject characteristics, random allocation, blindness between subjects and researchers, measurement and classification bias of results and evaluation measures, number of samples, analysis methods, variance estimate for major results, confounding variable control, result reporting, and conclusion) based on the criteria. Each item was measured using a three-point scale (Yes=2, Partial=1, No=0, N/A). The scores of all 14 items were summed and converted into percentages. The overall quality of the study was assessed as strong (> 80%), good (70%–80%), adequate (50%–69%), and limited (>50%). The quality assessment of studies was conducted by two researchers independently. When there was any discrepancy between the results (scores) of them, a consensus was drawn by discussion.

### Meta-Analysis

A meta-analysis was performed using R version 3.4.3. The data used in the analysis was generated by calculating the difference between means standardized by the mean difference and standard deviations of the control group and the treatment group. The mean difference standardized by the standard deviation was calculated by equation (1) .

$$\sqrt{S1_{\mathrm{pre}}^{2}+S1_{\mathrm{post}}^{2}-\left(2\times \mathrm{Corr}\times S1_{\mathrm{pre}}\times S1_{\mathrm{post}}\right)}$$

For effect size, standard mean difference (SMD) was analyzed by using Hedge’s g and the significance of effect size was confirmed in 95% confidence interval. Based on the criteria, when the estimated effect size was less than 0.32, it was ‘small effect’. When it was 0.33–0.55 or >0.56, it was considered ‘medium effect’ or ‘large effect’, respectively (20).

## Results

### Results of Quality Assessment

The results of the quality assessment of the literature showed that the score of the quality assessment on five studies ranged from 21 to 26 points, rated good or better (Table 1). All studies systematically presented “study objective”, “study design”, and “conclusion” and they were suitable for each item. The procedure of random assignment was described in the method section. All five studies blinded subjects but only three studies blinded researchers (11–13). While four studies (11, 13–15), except for one study (12), described measurement methods and evaluation tools in detail, all five studies did not calculate the number of samples before conducting the experiment. Although all studies reported “estimates of variance for key outcomes”, only three studies controlled confounding variables (11, 12, 15).

**Table 1:** Results of Quality Assessment

***Criteria Study***	***1***	***2***	***3***	***4***	***5***	***6***	***7***	***8***	***9***	***10***	***11***	***12***	***13***	***14***	***Total***
Cotelli, et al. 2014 (11)	+	+	+	+	±	+	+	+	±	+	+	+	+	+	26
Biundo, et al. 2015 (12)	+	+	+	±	±	+	+	±	±	±	+	+	+	+	23
André, et al. 2016 (13)	+	+	±	±	±	−	+	+	±	+	+	±	+	+	21
Roncero, et al. 2017 (14)	+	+	±	+	±	+	+	+	±	+	+	±	+	+	24
Lawrence, et al. 2018 (15)	+	+	+	+	+	−	−	+	±	+	+	+	+	+	23

+=2, ±=1, −=0

### Effects of tDCS on Patients with Cognitive Impairment in Old Age

The intervention effects of tDCS on patients with cognitive impairment in old age are presented in Table 2.

**Table 2:** The intervention effects of tDCS on patients with cognitive impairment in old age

***Study and design***	***Participants***	***Intervention***				***Assessment***	***Outcomes***
		***Stimulated region***	***tDCS***	***Sham tDCS***	***Stimulation session***		
Biundo et al. (12) Blinding & RCT design	PD-MCI(n=24) 1. tDCS(n=12) : age=69.1±7.6 2. sham(n=12) : age=72.3±4.1	Left dorsolateral prefrontal cortex	2mA 20min	The electrodes were placed in the same position as the real tDCS	16 session (4 d a week for 4 wk)	Assessment: baseline - after 4 week - follow up (6 week)	tDCS effect for naming and semantic fluency does not show significant difference from sham condition until follow-up period.
Lawrence et al. (15) RCT design	PD-MC I(n=42) 1. Standard cognitive training (n=7, analysis n=5) : age=68.14±8.69 2. tDCS(n=7) : age=72 ±6.45	Left dorsal lateral prefrontal cortex	1.5mA 20min	Control group received only neuropsychological assessments	4 wk	Assessment: baseline - post intervention - follow up (12 week)	All of the intervention groups showed no improvement in naming ability.
André et al. (13) Blinding & RCT design	VD(n=22) : age=63–94 1. tDCS(n=13) 2. sham(n=9, analysis n=8)	Left dorsal lateral prefrontal cortex	2mA 20min	8s	4 sessions (4 d)	Assessment: before tDCS - just after tDCS - follow up (2 wk)	Only the tDCS condition significantly improved scores from baseline to follow-up period.
Roncero et al. (14) Blinding & Crossover & RCT design	AD & FTD(n=10) : age=56–75 1. tDCS(n=5) 2. sham(n=5)	Left inferior parieto-temporal region	2mA 30min	60s	10 sessions	Assessment: baseline - after intervention - follow up	tDCS intervention group trained and untrained items score increased.
Cotelli et al. (11) Blinding & RCT design	AD(n=36) 1. AtDCS + IC memory training (n=12) : age=76.6 ± 4.6 2. PtDCS + IC memroy training (n=12) : age=74.7 ± 6.1 3. AtDCS + motor training (n=12) : age=78.2 ± 5.2	Left dorsal lateral prefrontal cortex	2mA 25min	10s	5 sessions	* Assessment: baseline - post intervention(2 wk) - follow up(3 months) - follow up (6 months) 1. Face-Name associations task (FNAT)	* Both groups that received IC memory training after intervention have increased FNAT accuracy compared to those that received motor training.

The combined effects of tDCS and cognitive training were examined on 24 Mild cognitive impairment in Parkinson’s disease (PD-MCI) patients and reported that naming and semantic fluency were not significantly different from the stimulation intervention group until the follow-up treatment (12). Similarly, tDSC did not improve the naming ability of subjects significantly after examining the effects of tDCS and the combined effects of tDCS and cognitive training (15).

On the other hand, the effects of tDCS intervention on patients with VD was evaluated and revealed that the placebo-stimulation condition did not improve naming ability significantly, whereas the naming performance of the tDCS intervention group significantly increased during the follow-up treatment compared to the baseline (13). Moreover, who the intervention effects on AD patients was tested for three groups (tDCS + memory training, placebo tDCS + memory training, and tDCS and physical exercise training), reported that the accuracy of the face-name associations task (FNAT) of the tDCS + memory training group increased significantly more than that of the tDCS + physical exercise training group (11).

## Results of Meta-Analysis

### Effects of tDCS Intervention on Naming Ability of Dementia Patients

**Fig. 2:**
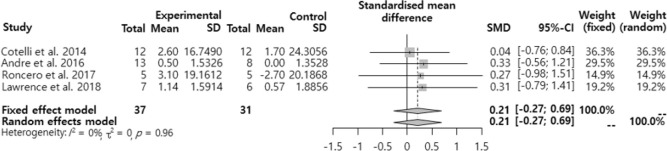
Effects of the tDCS intervention on the naming ability of dementia patients

The SMD regarding the effects of the tDCS intervention on the naming ability of dementia patients was analyzed (Fig. 2). The effect size was ‘small effect (SMD=0.21, 95% CI: −0.27, −0.69)’ and it was not significant at 95%. confidence interval.

### Combined Effects of tDCS + Language/Cognitive Training on the Naming Ability of Patients with Neurological Speech Disorder

**Fig. 3:**
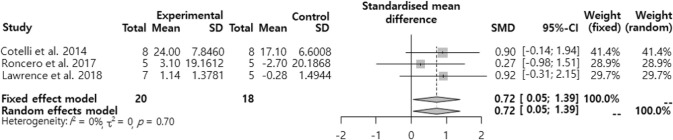
The combined effects of the tDCS intervention + language/cognitive training on the naming ability of dementia patients

The SMD regarding the combined effects of the tDCS intervention + language/cognitive training on the naming ability of dementia patients was analyzed (Fig. 3). The effect size of the tDCS intervention + language/cognitive training was significant ‘large effect (SMD=0.72, 95% CI: 0.05, 1.39)’.

## Discussion

This study conducted a quality assessment on five publications on the effects of tDCS on the naming ability to find that most of them were designed as a randomized controlled trial (RCT) but all five studies did not estimate the same size before conducting the experiments. Since the bias generated by sample sizes may pervert the results, future RCT studies to verify the effectiveness of tDCS are required to conduct a power test before conducting an experiment.

The results of this study’s meta-analysis showed that the effect of tDCS on the naming ability of dementia patients was not significant. It can be explained by two possibilities. The first is that the effects of tDCS on the improvement of naming ability were minimal. To date, previous studies on the effects of tDCS have reported conflicting results. Although many studies (11, 13, 14) have proved the effectiveness of tDCS, Biundo et al.(12) who evaluated the effects of tDCS on 24 PD-MCI patients showed that confrontational naming and semantic fluency were not significantly different from the placebo group. Moreover, tDCS did not significantly improve the naming ability (15).

tDCS is a therapeutic technology that stimulates the brain by flowing a DC current to the scalp. The general mechanism of tDCS is that, when minute DC current flows through the scalp, approximately 10%–20% of weak DC current reaches the cortex and it promotes or inhibits the spontaneous activity of the brain nerve. Since the brain is generally stimulated with a current below 2 mA, it does not induce an action potential but merely regulates the brain nerve. It affects the spontaneity discharge rate of nerve cells and the activation of N-Methyl-D-aspartic acid (NMDA) receptors (21) by regulating the resting membrane voltage of nerve cells (21). However, the mechanism of how tDCS influences the improvement of naming has not been clearly identified to date, and there is insufficient evidence for the long-term sustained effects of tDCS (22). However, regarding the effects of tDCS on cognitive functions such as memory, it may have a long-term sustained effect as well as a short-term sustained effect because of tDCS influences not only NMDA but also transmembrane potential difference (23). Nevertheless, long-term longitudinal studies are needed to identify the effects of tDCS in the future enough because studies examining the effectiveness of tDCS began to be accumulated in earnest since 2008 and there are only a few large-scale and long-term studies evaluating the improvement of naming owing to tDCS.

Secondly, the overall effects of tDCS on naming performance were not significant because the sample size of the analyzed studies was too small. In this meta-study, the effect size of the individual study and the overall effect size had positive signs coincidentally. However, the variance was relatively large due to the small sample size and the significance of the overall effect size was not accepted in a 95% confidence interval. Therefore, an additional meta-analysis including studies based on larger sample sizes is needed to scientifically prove the effectiveness of tDCS.

Another key finding of this study was that the combination of tDCS intervention and language/cognitive training made a significantly large impact. The combination of tDCS intervention and language/cognitive training may have a synergistic effect than a single intervention that only conducts cognitive training (11). Various experimental studies are needed to prove the combined effects of tDCS and language/cognitive training in the future.

This meta-analysis study is meaningful because it provides a scientific basis for examining the effect of tDCS on the naming ability of dementia patients. The limitations of the study were that first, this study did not include the results of studies written in other languages such as Chinese, French, and German because this study analyzed studies written in English only and second, these results should be generalized with special caution because we could not conduct a bias test as less than 10 publications were analyzed and the variances and standard deviations of these studies were large.

## Conclusion

The combination of tDCS and language/cognitive training significantly improved the naming ability of dementia patients. Since the generalization of the results is limited due to small sample sizes of analyzed studies, it will be necessary to evaluate the effects of tDCS intervention using large-sample RCT studies that estimate the sample size in advance.

## Ethical considerations

Ethical issues (Including plagiarism, informed consent, misconduct, data fabrication and/or falsification, double publication and/or submission, redundancy, etc.) have been completely observed by the authors.
